# Healthy eating among people on opioid agonist therapy: a qualitative study of patients’ experiences and perspectives

**DOI:** 10.1186/s40795-024-00880-8

**Published:** 2024-05-05

**Authors:** Einar Furulund, Karl Trygve Druckrey-Fiskaaen, Siv-Elin Leirvåg Carlsen, Tesfaye Madebo, Lars T. Fadnes, Torgeir Gilje Lid

**Affiliations:** 1 Centre for Alcohol and Drug Research, Stavanger University Hospital, Stavanger, Norway; 2 Department of Addiction Medicine, Bergen Addiction Research, Haukeland University Hospital, Bergen, Norway; 3 Department of Global Public Health and Primary Care, University of Bergen, Bergen, Norway; 4 Department of Respiratory Medicine, Stavanger University Hospital, Stavanger, Norway; 5 Department of Clinical Science, University of Bergen, Bergen, Norway

**Keywords:** Diet, Vegetables, Fruit, Habits, Lifestyle, Substance-related disorders, Therapeutics, Methadone, Behavior and behavior mechanisms

## Abstract

People with substance use disorders often have unhealthy diets, high in sweets and processed foods but low in nutritious items like fruits and vegetables, increasing noncommunicable disease risks. This study investigates healthy eating perceptions and barriers among individuals with opioid use disorder undergoing opioid agonist therapy. Interviews with 14 participants at opioid agonist therapy clinics in Western Norway, using a semi-structured guide and systematic text condensation for analysis, reveal that most participants view their diet as inadequate and express a desire to improve for better health. Barriers to healthy eating included oral health problems, smoking habits, and limited social relations, while economic factors were less of a concern for the participants. Participants did find healthy eating easier when they were in social settings. This study underscores the importance of understanding and addressing these barriers and facilitators to foster healthier eating patterns in this population, potentially enhancing overall health and well-being.

**Supplementary Information:**

The online version contains supplementary material available at 10.1186/s40795-024-00880-8.

## Introduction

Substance use disorder (SUD), particularly opioid use disorder (OUD), is complex and extends beyond the risk of overdose, suicide, and infection. Noncommunicable diseases, such as chronic lung diseases, cancer, and cardiovascular diseases, all contribute to increased morbidity and mortality [1, 2]. Nutrition is an important but often overlooked aspect of SUD recovery [3]. Individuals, particularly those with OUD, often report unhealthy eating habits consisting of a high consumption of sweets, sugar-sweetened and processed foods and a low consumption of fruits and vegetables [4–8]. Comorbidities might arise or worsen because of an unhealthy eating behavior [9, 10]. Furthermore, substance use can severely impact an individuals’ nutritional habits and diet, as substances are often favored over food [11]. According to recent Norwegian research [12, 13], approximately half of the patients receiving opioid agonist therapy (OAT) are deficient in vitamin D and folic acid.

Despite the established link between dietary habits and health outcomes in the general population, few studies have focused on nutritional interventions for populations with SUD and OUD [14]. A diet containing a higher intake of fruits and vegetables may reduce morbidity in high-risk populations by improving cardiovascular and mental health, as well as biomarkers of cellular stress defense [15–18]. However, it is unclear to what extent interventions aimed at other high-risk groups could be applied directly to OAT patients. This research gap highlights the need for improved understanding of dietary behaviors and attitudes to healthy eating among individuals in OAT.

## Materials and methods

This study aims to provide insights into the dietary habits and views of healthy eating among individuals with opioid use disorders receiving opioid agonist therapy. In addition, we intend to lay the framework for the development of dietary interventions that could improve health outcomes and quality of life for individuals receiving OAT by identifying barriers and facilitators.

### Design

ATLAS4LAR project aims to improve the health and well-being of individuals with opioid use disorder undergoing OAT [19]. The project enrols OAT patients from Stavanger and Bergen, two cities in Western Norway, into a cohort and a health registry. This article was based on participants in this cohort. A semi-structured interview guide on dietary habits and perceptions of and barriers to healthy eating was developed as a collaboration between the study group, research nurses, clinicians, and user representatives. The interview guide covers topics of physical activity, smoking cessation [20] and healthy eating; this article focuses on healthy eating. A COREQ checklist [21] was applied and is included in the supplementary file.

### Study sample and setting

Interviews were conducted with 14 patients at OAT clinics in Stavanger and Bergen, the two largest cities in Western Norway. All patients who completed an annual health assessment and willing to complete an interview about lifestyle were eligible to participate in the study. There were no specific exclusion criteria. Most patients receive follow-up on a weekly basis from multidisciplinary teams, including monitoring of OAT medication intake such as buprenorphine and methadone. For more information regarding the included outpatient clinics, see Fadnes et al. (2019) [22]. The research nurses collaborated with OAT clinicians to recruit a purposive sample from four different clinics in Bergen and Stavanger. Our goal was to recruit participants from various OAT clinics and ages and genders; the study sample characteristics are outlined in Table 1.

### Data collection

Among the 14 participants, thirteen completed the full interview guide, with one participant leaving the interview after twelve minutes with an incomplete interview. All the 14 participants consented to the interviews being audio recorded. All interviews were conducted during the ongoing COVID-19 pandemic in January and February 2021. Necessary precautions were taken to minimise the risk of transmitting viruses during the interview. This included symptom checklists for COVID-19, maintaining distance, and occasionally wearing facemasks. Three research nurses with training in qualitative interviewing contacted patients by phone or when they had an appointment at the clinic and conducted the interviews. They were instructed to move between topics and questions based on interview dynamics. The final interview guide included three nutrition-related issues: (1) reflections on their daily diets, (2) opportunities to prioritise healthy eating in their daily lives, and (3) reflections on the need to change their diets. See the supplementary file for the interview guide. A total of forty to sixty minutes were spent on each interview.

### Data analysis

Due to COVID-19 and geographical distances between researchers, we conducted our meetings through Microsoft Teams for video conferencing and used NVivo 20 for the data analysis A pseudonym reflecting the gender of the participants was assigned to each recording, and it was transcribed verbatim by the study’s authors (EF, SELC, and KTDF). The analysis followed the four steps of systematic text condensation [23, 24]. At first, the authors spent extensive time reading the transcripts to better understand what was being said. This thorough reading led to identifying preliminary themes, their presentation, and a collaborative discussion in a workshop. As a result of this discussion, some central themes were agreed upon for further analysis. Afterwards, a second reading was conducted to identify meaningful units, which were then categorized under the earlier themes. The lead author led the data analysis in close collaboration with SELC, KTDF, and TGL. TM and LTF also contributed significantly, ensuring a collective analysis effort. This collaborative approach facilitated the generation of condensed versions that captured the essence of the categorized themes. Ongoing discussions on terminology and limitations among all co-authors ensured clarity and coherence throughout the process. In the end, these condensed insights formed the basis of an overall narrative that addressed the aim of the study.

## Results

14 participants were interviewed, 11 male and three females, and all receiving OAT (Table 1). All participants had relatively stable housing conditions, and six lived alone. Five had injected drugs within the past six months before the interview. Thirteen reported smoking at least three times a week. The median debut age for tobacco, alcohol and cannabis was 13 to 14 years, while for stimulants it was 23 years and for opioids 25 years.

**Table 1:** Characteristics of the participants

Age, median (range)	49 (30–60)
Female/male	3 / 11
OAT medication, n	
*Methadone*	4 of 14
*Buprenorphine*	10 of 14
Education	
*Incomplete primary education* ^*1*^	3 of 14
*Completed primary education* ^*1*^	5 of 14
*High school*	4 of 14
*University*	2 of 14

^1^ The first ten years of school are mandatory in Norway.

In this analysis, the researches extracted three themes and several subthemes reflecting the complex interactions between personal health, social environment, and dietary practices. For instance, the theme “Dietary Patterns and Health Practices” explored varied dietary habits among participants, from structured meals incorporating traditional Norwegian foods to periods of unhealthy eating dominated by fast foods and convenience items. Sub-themes include the impact of drug use on dietary habits and the role of smoking in influencing taste and appetite. The theme “Barriers and facilitators to healthy eating” discussed factors influencing patients’ ability to maintain a healthy diet, including economic constraints, access to cooking facilities, and treatment facilities’, and physical and social environment. Sub-themes highlighted the role of oral health in dietary choices and the potential of nutritional interventions within OAT clinics. The last theme, “Social and psychological dimensions of eating”, addressed the social context, focusing on how living arrangements and social interactions influence dietary choices. This theme also delves into the stigma associated with substance use and its impact on participants’ nutritional choices and self-perception.

Participants differed greatly in their eating patterns. Most participants acknowledged the importance of increasing fruit and vegetable consumption and expressed a wish to eat healthier. Some perceived their diet as well-balanced, which included multiple meals of traditional Norwegian foods. Others reported having unstable dietary habits, expressed as having healthy periods of eating nutritious foods, and less healthy periods with mainly intake of unhealthy foods such as fast foods. Additionally, some said they almost did not eat for long periods. Some participants felt they needed more knowledge to implement a nutritious diet into their daily lives.

### Economy and access to a kitchen were not important barriers to healthy eating

Although most participants said they could afford healthy food and maintain a healthy diet, some highlighted that they could not afford high-priced food like fresh fish or meat several times a week. Nevertheless, it was possible to cook nutritious food despite having little money. Some participants also mentioned vitamin supplements as a means of enhancing their nutrition.

“Yes, I want to eat healthier food. Much of what is healthy is not that expensive. Buy some tomatoes, cucumber, lemon, and salad. Then, we look for where there is an offer, and we go to each store and pick what is on offer”. - Thomas.

For many participants, the kitchen was a space of both opportunity and challenge. While some engaged in regular meal preparations, others found themselves limited to heating pre-processed foods. Living in treatment facilities posed challenges due to their strict schedules and predetermined diets. Some participants had experienced being responsible for prepare food for the institution and other patients. These routines could be quite demanding, and they could become tired of cooking. An interesting introduction to smoothies was noted in some substance use treatment facilities, for making smoothies accessible where the institutions did buy the fruits and vegetables and stood with available equipment. This was without any cost to the patients. The participants expressed appreciation for the smoothies, citing their taste and feeling healthy as key reasons for the positive reception. After discharge from these institutions, none of the participants regularly continued to make or purchase smoothies.

“If you live in an institution or in those places where you are not completely in charge and do not have your own apartment, then it is probably more difficult to inspire yourself to cook and eat healthy …” - Thomas.

### Struggling with stigma related to substance use

Several participants knew of food distribution centres that provided free food. Some said it was a helpful initiative to distribute free food to people in need. In contrast, others experienced barriers such as the stigma of being seen at these centres, or the risk of meeting people under the influence of drugs.

“And I do not like going to those Salvation Army [having a food provision service] centres, because I meet so many weird people [trying to sell drugs] … It can be tough to say no [offers for drugs] to those people sometimes”. - Erik.

Some participants mentioned the drug-related stigma linked to low weight. Some participants did not view their weight as crucial to their overall well-being. However, a few participants reported that their family members focused on the participant’s weight and associated this with their life situation, specifically their substance use.

“… about the kilos. It is not something like that, I think I’m very thin or something like that, but I hear from family members that I have now lost weight. Then, I know that they associate it with illicit drugs and that things are not going well. That probably affects me more than just those kilos”. - Kristian.

Some participants stated that they struggled to gain weight, even though they wanted a better appetite to increase their body weight. Some also made choices accordingly, such as eating a high-fat diet. Despite this, weight gain remained a constant struggle. While some participants had specific goals to increase their body weight by five to ten kilograms, they faced challenges in achieving this in a healthy manner. Despite their intention to gain weight, the participants expressed concerns about excessive sugar intake and its impact on their overall health. The struggle to balance weight gain with a nutritious diet and a lack of self-confidence in the kitchen made it difficult for them to adhere to balanced and healthy eating.

“To gain more weight, I try to eat fat-rich foods. Yes, it is the usual routine with breakfast, lunch and dinner, and there are also snacks in between, and of course, then I eat supper”. - Peter.

### Oral health status and smoking impact negatively on healthy eating

Poor oral health was a major barrier that greatly impacted the participants’ diet. Missing several teeth, poorly adapted dentures and pain in their mouth restricted many from eating many of the fruits and vegetables. Some described hard fruits and vegetables such as apples and carrots as impossible for them to eat. Participants with poorly adapted dentures expressed difficulties in eating and needing to clean their dentures after eating, which were perceived as embarrassing and stigmatising in social settings.

“Meat, yes, and then it gets stuck. So, I always have to take [the denture] out after I finish eating. Then, I need to go to rinse my mouth. It was not how I imagined it when I got it [denture]…. The only thing I have been able to chew is bananas and oranges, because they are soft”. - Jacob.

Several participants reported that smoking negatively impacted their taste, reduced their appetite of food, and affected their daily food consumption. Smokers who reduced or stopped smoking, experienced an increase in appetite and a positive impact on the taste of food.

“When I stopped smoking, my taste returned to normal, and my appetite improved since smoking “killed” some of my taste for food”. - Thomas.

### The social context is important for all aspects of eating

Most participants expressed that food has a social function, particularly among those who live alone. Participants who had cohabitants also said their diet would have been negatively affected if they had lived alone. Establishing or maintaining healthy eating habits were challenging to many who regularly were eating alone. Some participants did not see the value of making an entire meal just for themselves. Furthermore, when participants were alone, purchasing unhealthy foods such as doughnuts and fast food was easier.

“I see I have such a good diet only because I live with someone. It is better to be two people eating together rather than alone… yes, it has a lot to say. Many complain and say exactly that [to me]; ‘you are lucky to be two people’ [eating together]”. - Jacob.

Some participants found that creating a shopping list simplified the grocery shopping process. Although many lacked the discipline to organize a shopping list and preferred not to shop alone, they found it more manageable to shop with family or friends. Alone, participants said to buy unhealthy food, high in fat, and sugar. In contrast, shopping with others often led to healthier choices. However, for a few participants, the challenge was not in purchasing nutritious food but in the actual cooking and preparation of meals.

“I go to the store every other day to buy food. Instead of thinking ahead that tomorrow I will have this for dinner, and then I will have that for dinner the next day”, I make a list like that in my head. However, I do this [buys the food], and the food ends up in the freezer, and then it stays there”. - Oliver.

### Preferences relevant to nutritional interventions in the clinic

We specifically asked participants about their preferences for establishing nutritional interventions in their OAT clinic. Most participants wanted to consume more fruits and vegetables, recognising their health benefits and appealing taste. They thought the OAT clinics should promote a healthy diet more actively, e.g., with posters in the waiting room. These posters could include basic information about different foods and about the consequences of not eating healthy. Other suggestions were more extensive, with a clinic-initiated patient-oriented educational cooking programme, focusing on easy recipes of affordable and tasty food.

## Discussion

The study offers fresh insights into the viewpoints and choices of patients regarding healthy eating within the context of OAT. Numerous participants highlighted challenges such as oral health concerns, smoking habits, and reduced social interactions that hinder their ability to adhere to a healthy diet. Interestingly, economic constraints were cited by only a minority of participants as barriers. Additionally, some individuals expressed that they found it easier to sustain a healthy diet when they had social support and stressed the importance of having a structured grocery shopping list.

It is essential to acknowledge that several factors play a role in dietary choices among the OAT population [25]. Our research has shown a notable shift in the average age of individuals in our sample compared to previous studies. This demographic transformation toward an older population is associated with an increased susceptibility to chronic diseases and a higher risk of malnutrition [26]. Specifically, the average age of patients undergoing Opioid Agonist Therapy (OAT) now stands at 47, with a median age of 49 within our dataset. A decade ago, the typical age for this OAT patient group was 42, marking a significant increase of five years over the past decade. Notably, the proportion of OAT patients aged 60 and above has tripled in 2021 when compared to data from 2015 [27]. Furthermore, within our sample, we have observed significant variations in dietary habits and meal frequency among this demographic. Nevertheless, in the context of an ageing population, the importance of adopting healthy eating habits becomes even more pronounced to reduce the risk of chronic diseases, including cardiovascular and metabolic disorders.

In Norway, people with SUD have a high level of social support offered by the government in terms of financial help, free or subsidised health care services, and mostly stable living conditions [28]. Previous studies have found that people with long-term SUD experience economic challenges and unstable living conditions, making it difficult to achieve adequate nutrition [4, 29]. Our participants, however, did not see the economy as a primary obstacle to healthy eating. Despite some financial limitations, such as the need to prioritise and adhere to a budget, they were generally able to afford several healthy foods as components for their diets. Interestingly, some participants who lived together with others said they were always looking for reasonable offers on food at the store, almost like a sport. This may support that they do not necessarily have a stable and strong economy but have strategies to manage economic limitations. However, even with a stable living situation and kitchen access, they still found attaining a healthy diet to be difficult.

Poor oral health significantly hinders the ability to maintain a nutritious diet primarily due to missing teeth, oral pain, or dentures. Many participants expressed how their oral health directly influenced their diet. These physical constraints and the potential for social discomfort provide insights into the infrequent consumption of fruits and vegetables, even when individuals are aware of their nutritional benefits. This aligns with findings on older adults [30], with many reporting poor oral health due to lacking and damaged teeth. As a result, they have fewer choices for food, such as fruits, vegetables, and fibre, increasing their risk of unhealthy food choices [30]. However, not all the participants in our study described difficulties eating healthy food. Interestingly, some were introduced to smoothies at treatment facilities and found them appealing. After being discharged, however, they did not continue to make or purchase smoothies.

A large study found an inverse relationship emerged between cigarette smoking and eating healthy food. Specifically, as individuals increase their daily smoking, their intake of healthy foods, such as fruits and vegetables, declined. This is in line with our findings, where participants shared that smoking adversely affected their ability to taste the food [8]. This effect may be attributed to nicotine’s widely recognized capacity to suppress appetite, potentially prompting individuals to turn to smoking as a substitute behavior for eating [31].

The absence of individuals to share meals with and feelings of loneliness posed a significant obstacle to the participants’ attempts to sustain a nutritious diet. Many conversations centred around the challenges of grocery shopping and meal preparation for solitary individuals. These observations align with previous research, which has shown that people living alone typically consume fewer fruits, vegetables, and fish compared to those who have meal companions [32]. Although our data do not allow direct comparisons, the narratives from our participants are consistent with these findings. In contrast, those living with others credited their dietary stability to their shared living arrangements. They believed that having someone to share meals with added an extra layer of meaning and purpose to meals.

Many participants were interested in adopting healthier eating habits, a positive and noteworthy finding considering their substance use history. This shift towards healthier diets may indicate an awareness of the correlation between health and nutrition even after prolonged substance use. These individuals recognized the potential for an improved diet to enhance their health. Some associated a healthy diet with weight gain as a sign that their drug problem is under control. Those who wanted to increase their weight said they needed to pay attention to their diet, which could be exhausting. A number of epidemiological studies have investigated the relationship between drug use and body weight, and most of the evidence demonstrates an inverse correlation [33]. A regular diet can have therapeutic benefits, including improving health, self-esteem and social relationships [25].

During the interview, participants were asked for intervention preferences relevant to their dietary and nutritional needs. Interestingly, not all participants came up with specific suggestions for this topic, yet some proposed the idea of making information available in the waiting rooms or initiating cooking courses. As an alternative, some participants suggested that smoothies would be beneficial to consume more fruit and vegetables without damaging their teeth. The potential of smoothies as effective dietary interventions has been explored in different populations, including adolescents in schools and older adults [34, 35].

The current study has several strengths and limitations. The qualitative design provides an in-depth understanding of participants’ experiences, and of barriers and facilitators to healthy eating. However, one limitation regarding its design is that nutritional status was not measured in our study. The participants described their nutritional status through their eating habits, detailing their behaviour and experiences associated with eating patterns. Research nurses assisted with providing insights into how to perform the interview and the interview guide on how to phrase the questions in an understandable way. User representatives offered valuable insight to ensure the relevance and highlighted cultural and societal factors. Even though research nurses are separate from the clinical care, bias may still occur. Some patients may be more inclined to present themselves rather than express their feelings or give feedback. Through collaboration in frequent digital meetings and using a theoretical framework, we were able to test ideas and interpretations, and thus reduce the influence of investigator bias [36]. It is likely that social desirability influenced the interview process and results. When data were collected, the study participants were receiving treatment from OAT outpatient clinics where they were interviewed, which may have made them more susceptible to social desirability bias during interviews [37]. The interview guide was designed to minimise such bias, as well as the choice of interviewer being a research nurse and not their contact person/clinician. The participants could steer the order of the topics in the discussion, which probably enabled them to speak more freely from their perspectives.

Together with earlier work, this study emphasises the importance of understanding patients’ perspectives and needs regarding nutrition [25, 38]. According to patients, diet and nutrition are important and bidirectionally interlinked with their substance use. Healthcare providers should address the diet and nutrition of patients to facilitate recovery. However, strategies to improve oral health among OAT patients, and motivational and educational strategies to improve cooking skills, are necessary prerequisites in addition to the more specific interventions, to improve patients’ recovery and their overall health.

The OAT platform facilitates communication between healthcare professionals and hard-to-reach patients. To prioritize nutrition, five key topics have been proposed: incorporating discussions about food and nutrition history into clinical consultations, conducting anthropometric measurements including regular weight monitoring, utilizing biochemical data to identify dietary limitations, evaluating potential health implications of individuals’ nutritional profiles, and tailoring approaches based on clients’ personal histories and perspectives [39]. The results indicate that a combination of individual, social, and environmental factors influenced participants’ dietary habits and eating patterns.

## Conclusions

In conclusion, our findings shed light on several critical aspects of a healthy diet among patients in OAT. Oral health issues, smoking habits, and limited social interaction emerged as significant impediments to upholding a nutritious diet. Healthcare professionals should proactively tackle these obstacles, while future research should prioritize devising effective strategies to overcome these barriers and improve the dietary patterns, nutritional well-being, and overall health of individuals undergoing OAT.

### Electronic supplementary material

Below is the link to the electronic supplementary material.

Supplementary Material 1

Supplementary Material 2

## Data Availability

Because of data protection regulations, the raw interview data for this study are not publicly available.

## Abbreviations

SUD: Substance use disorder
OUD: Opioid use disorder
OAT: opioid agonist therapy
COREQ: Consolidated criteria for reporting qualitative research

## References

[1] Hser YI, Mooney LJ, Saxon AJ, Miotto K, Bell DS, Zhu Y (2017). High mortality among patients with opioid Use Disorder in a large Healthcare System. J Addict Med.

[2] Lewer D, Jones NR, Hickman M, Nielsen S, Degenhardt L (2020). Life expectancy of people who are dependent on opioids: a cohort study in New South Wales, Australia. J Psychiatr Res.

[3] Whatnall MC, Skinner J, Pursey K, Brain K, Collins R, Hutchesson MJ, Burrows TL (2021). Efficacy of dietary interventions in individuals with substance use disorders for illicit substances or illicit use of pharmaceutical substances: a systematic review. J Hum Nutr Diet.

[4] Sæland M, Haugen M, Eriksen FL, Wandel M, Smehaugen A, Böhmer T, Oshaug A (2011). High sugar consumption and poor nutrient intake among drug addicts in Oslo, Norway. Br J Nutr.

[5] Saeland M, Wandel M, Böhmer T, Haugen M (2014). Abscess infections and malnutrition – a cross-sectional study of polydrug addicts in Oslo, Norway. Scand J Clin Lab Investig.

[6] Nolan LJ, Scagnelli LM (2007). Preference for sweet foods and higher body mass index in patients being treated in long-term methadone maintenance. Subst Use Misuse.

[7] Sæland M, Haugen M, Eriksen FL, Smehaugen A, Wandel M, Böhmer T, Oshaug A (2009). Living as a drug addict in Oslo, Norway – a study focusing on nutrition and health. Public Health Nutr.

[8] Morabia A, Curtin F, Bernstein MS (1999). Effects of smoking and smoking cessation on dietary habits of a Swiss urban population. Eur J Clin Nutr.

[9] Willett W, Rockström J, Loken B, Springmann M, Lang T, Vermeulen S (2019). Food in the Anthropocene: the EAT–Lancet Commission on healthy diets from sustainable food systems. Lancet.

[10] Hendricks K, Gorbach S (2009). Nutrition issues in chronic drug users living with HIV infection. Addict Sci Clin Pract.

[11] Nabipour S, Ayu Said M, Hussain Habil M (2014). Burden and nutritional deficiencies in opiate addiction- systematic review article. Iran J Public Health.

[12] Bemanian M, Chowdhury R, Stokke K, Aas CF, Johansson KA, Vold JH, Fadnes LT (2022). Vitamin D status and associations with substance use patterns among people with severe substance use disorders in Western Norway. Sci Rep.

[13] Bemanian M, Vold JH, Chowdhury R, Aas CF, Gjestad R, Johansson KA, Fadnes LT. Folate Status as a Nutritional Indicator among people with Substance Use Disorder; a prospective cohort study in Norway. Int J Environ Res Public Health. 2022;19(9). 10.3390/ijerph19095754 PMC9099634 35565159

[14] Mahboub N, Rizk R, Karavetian M, de Vries N (2021). Nutritional status and eating habits of people who use drugs and/or are undergoing treatment for recovery: a narrative review. Nutr Rev.

[15] Furulund E, Bemanian M, Berggren N, Madebo T, Rivedal SH, Lid TG, Fadnes LT (2021). Effects of Nutritional interventions in individuals with chronic obstructive lung disease: a systematic review of Randomized controlled trials. Int J Chron Obstruct Pulmon Dis.

[16] Jacka FN, O’Neil A, Opie R, Itsiopoulos C, Cotton S, Mohebbi M (2017). A randomised controlled trial of dietary improvement for adults with major depression (the ‘SMILES’ trial). BMC Med.

[17] Bøhn SK, Myhrstad MC, Thoresen M, Holden M, Karlsen A, Tunheim SH (2010). Blood cell gene expression associated with cellular stress defense is modulated by antioxidant-rich food in a randomised controlled clinical trial of male smokers. BMC Med.

[18] Aune D, Giovannucci E, Boffetta P, Fadnes LT, Keum N, Norat T (2017). Fruit and vegetable intake and the risk of cardiovascular disease, total cancer and all-cause mortality—a systematic review and dose-response meta-analysis of prospective studies. Int J Epidemiol.

[19] Fadnes LT. ATLAS4LAR: Kartlegging og behandling av lungesykdom i legemiddelassistert behandling 2019 [updated 18.12.2019; cited 2020 21.09.2020]. https://helse-bergen.no/avdelinger/rusmedisin/rusmedisin-seksjon-forsking/bar/atlas4lar-kartlegging-og-behandling-av-lungesykdom-i-legemiddelassistert-behandling.

[20] Druckrey-Fiskaaen KT, Furulund E, Madebo T, Carlsen S-EL, Fadnes LT, Lid TG (2023). A qualitative study on people with opioid use disorders’ perspectives on smoking and smoking cessation interventions. Front Psychiatry.

[21] Tong A, Sainsbury P, Craig J (2007). Consolidated criteria for reporting qualitative research (COREQ): a 32-item checklist for interviews and focus groups. Int J Qual Health Care.

[22] Fadnes LT, Aas CF, Vold JH, Ohldieck C, Leiva RA, Chalabianloo F (2019). Integrated treatment of hepatitis C virus infection among people who inject drugs: study protocol for a randomised controlled trial (INTRO-HCV). BMC Infect Dis.

[23] Malterud K (2012). Systematic text condensation: a strategy for qualitative analysis. Scand J Public Health.

[24] Malterud K (2011). Kvalitative metoder i medisinsk forskning: en innføring. 3. Utg.

[25] Neale J, Nettleton S, Pickering L, Fischer J (2012). Eating patterns among heroin users: a qualitative study with implications for nutritional interventions. Addiction.

[26] Norman K, Haß U, Pirlich M. Malnutrition in older adults-recent advances and remaining challenges. Nutrients. 2021;13(8). 10.3390/nu13082764 PMC8399049 34444924

[27] Bech ABBA, Lobmaier P, Skeie I, Lillevold PH, Clausen T, SERAF Statusrapport LAR. 2021. University Oslo, Center for addiction research; 2022. Report No.: 2/2022.

[28] Health Mo, Services C. Se Meg! En Helhetlig Rusmiddelpolitikk, Alkohol–Narkotika–Doping [See me! An overall policy for Alcohol, drugs and Doping]. Helse-og omsorgsdepartementet Oslo; 2012.

[29] Himmelgreen DA, Pérez-Escamilla R, Segura-Millán S, Romero-Daza N, Tanasescu M, Singer M (1998). A comparison of the nutritional status and food security of drug-using and non-drug-using hispanic women in Hartford, Connecticut. Am J Phys Anthropol.

[30] Toniazzo MP, Amorim PSA, Muniz FWMG, Weidlich P (2018). Relationship of nutritional status and oral health in elderly: systematic review with meta-analysis. Clin Nutr.

[31] Audrain-McGovern J, Benowitz NL (2011). Cigarette smoking, nicotine, and body weight. Clin Pharmacol Ther.

[32] Hanna KL, Collins PF (2015). Relationship between living alone and food and nutrient intake. Nutr Rev.

[33] Li J, Yang C, Davey-Rothwell M, Latkin C (2016). Associations between Body Weight Status and Substance Use among African American Women in Baltimore, Maryland: the CHAT study. Subst Use Misuse.

[34] Bates D, Price J (2015). Impact of Fruit smoothies on adolescent Fruit Consumption at School. Health Educ Behav.

[35] Zhang JY, Lo HC, Yang FL, Liu YF, Wu WM, Chou CC. Plant-Based, antioxidant-rich snacks elevate plasma antioxidant ability and alter gut bacterial composition in older adults. Nutrients. 2021;13(11). 10.3390/nu13113872 PMC8624639 34836127

[36] Shenton AK (2004). Strategies for ensuring trustworthiness in qualitative research projects. Educ Inform.

[37] Latkin CA, Edwards C, Davey-Rothwell MA, Tobin KE (2017). The relationship between social desirability bias and self-reports of health, substance use, and social network factors among urban substance users in Baltimore, Maryland. Addict Behav.

[38] Matthews H, Diamond JB, Morrison D, Teitelbaum SA, Merlo LJ. Patient experiences with tobacco use during substance use disorder treatment and early recovery: a mixed method analysis of phone interview responses. J Addict Dis. 2022:1–7. 10.1080/10550887.2022.2103352 35930400

[39] Chavez MN, Rigg KK (2020). Nutritional implications of opioid use disorder: a guide for drug treatment providers. Psychol Addict Behav.

