# Supplementary material for: Healthy eating among people on opioid agonist therapy: a qualitative study of patients’ experiences and perspectives
Source: BMC Nutr. 2024 May 5;10:70. doi: 10.1186/s40795-024-00880-8 (PMC11071228; doi:10.1186/s40795-024-00880-8)
Supplement: Supplementary file 2 — Supplementary Material 2 [file 40795_2024_880_MOESM2_ESM.pdf]

English version

Supplementary file

Interview guide

Q1: What are your thoughts on your health today?

Q2: What do you think affects your health?

Topic: Diet and nutrition

Q3: What are your thoughts on your diet today?

Q4: Can you tell me a bit about your usual food and drink intake?

Q5: How many meals do you have per day?

Q6: Do you often cook for yourself? If no: why not?

Q7: Do you have access to a kitchen?

Q8: What types of food do you prepare?

Q9: What opportunities do you have to prioritize your diet?

Q10: How does your financial situation affect the type of food you buy?

Q11: Do you have any issues that make it difficult to eat?

Q12: If you were to change something about your diet, what would it be?

Q13: We want to start a project to help OAT patients have a healthier diet. What do you think such a project should offer?

Closing question

Is there anything else you would like to add to what we have talked about today?
